# Water Dimer-Driven DNA Base Superstructure with Mismatched Hydrogen Bonding

**DOI:** 10.1021/jacs.2c09575

**Published:** 2022-10-27

**Authors:** Shuning Cai, Lauri Kurki, Chen Xu, Adam S. Foster, Peter Liljeroth

**Affiliations:** † Department of Applied Physics, Aalto University, 00076 Aalto, Espoo, Finland; ‡ WPI Nano Life Science Institute (WPI-NanoLSI), Kanazawa University, Kakuma-machi, Kanazawa 920-1192, Japan

## Abstract

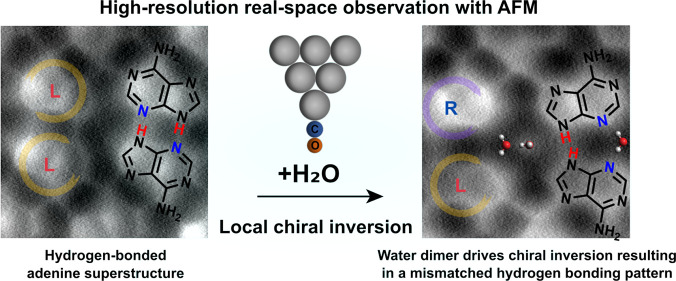

The existence of water dimers in equilibrium water vapor at room temperature and their anomalous properties revealed by recent studies suggest the benchmark role of water dimers in both experiment and theory. However, there has been a limited observation of individual water dimers due to the challenge of water separation and generation at the single-molecule level. Here, we achieve real-space imaging of individual confined water dimers embedded inside a self-assembled layer of a DNA base, adenine, on Ag(111). The hydration of the adenine layers by these water dimers causes a local surface chiral inversion in such a way that the neighboring homochiral adenine molecules become heterochiral after hydration, resulting in a mismatched hydrogen-bond pattern between neighboring adenine molecules. Furthermore, the mutual influence between the adenine superstructure and these dynamic confined water dimers is corroborated by theoretical simulation and calculations. The observation of single confined water dimers offers an unprecedented approach to studying the fundamental forms of water clusters and their interaction with the local chemical environment.

The ubiquitous character of water as a solvent in nature means that, in most cases, reactants and products are inevitably exposed to water and it plays a key role in the biochemical processes of living organisms. Intriguingly, many recent studies have expanded the role of water beyond a passive matrix to an active promoter,^1−3^ in chemical reactions^4−6^ and functional materials.^7−9^ In particular, water dimers not only are a fundamental unit for studying the properties of water but also show some exotic properties, such as an anomalously low barrier for diffusion on a surface involving nuclear quantum effects.^10−12^ Besides, water dimers have also been proved to be potential bifunctional catalysts^13,14^ and play a significant role in the initial stage of the ice nanocluster formation during the bilayer ice growth.^15−17^ However, separating and generating individual water dimers stabilized at ambient temperature remains challenging.^18^ Furthermore, many experimental techniques suffer from limited spatial resolution, yield ensemble-averaged results, or require complicated modeling to extract structural data. Thus, high-resolution structural data on the single-water-molecule level^19−22^ would significantly improve our understanding of water dimers.

A viable route to directly study the properties of water dimers takes advantage of the confinement possibilities offered by molecular assemblies. Supramolecular networks held together by non-covalent interactions have been considered as ideal models to gain insights into micro-hydration,^23,24^ due to their sensitivity to the external environment and ability to provide dynamic confinement. More generally, the extensive hydrogen bond (H-bond) is one of the most significant properties of water, and recently the role of confined water in DNA bases^25−27^ has aroused a broad interest in the investigation of DNA-related biological processes in vivo, creating novel opportunities in DNA nanotechnology. DNA bases record the genetic information on life through the H-bonded pairing mechanism with high efficiency and precision, which is widely applied in patterning diverse materials such as carbon nanotubes^28,29^ and enriching the crystal structures of nanoparticles.^30,31^ As one of the four nucleobases, adenine and its derivatives perform multiple functions in biochemistry. On the surface, adenine molecules form a H-bond-assisted 2D supramolecular network,^32^ which can be considered as a low-dimensional crystal,^33^ providing a unique platform to observe a micro-hydrated environment.

In this work, using low-temperature scanning tunneling microscopy (STM) and non-contact atomic force microscopy (nc-AFM) with a CO tip,^34,35^ we achieve real-space imaging of water dimers within a dynamic 2D adenine layer on Ag(111). Instead of desorption or randomly distributed clustering, these dimers form spontaneously at room temperature (RT). As demonstrated in Scheme 1, the introduction of water dimers induces a local surface chiral inversion^36^ in such a way that the neighboring homochiral adenine pairs become heterochiral (left and right handedness marked by the purple and orange circular arrows, respectively), accompanied by the emergence of a counter-intuitive mismatched H-bond structure (depicted by a red dashed circle). The necessity of the water dimer with a linear non-planar configuration in stabilizing the mismatched H-bonded superstructure is further confirmed by density functional theory (DFT) calculations. This direct observation provides rare evidence of the potential role of individual water dimers in self-assembly, paving the way for exploring the novel properties and future applications of water dimers.

**Scheme 1:**
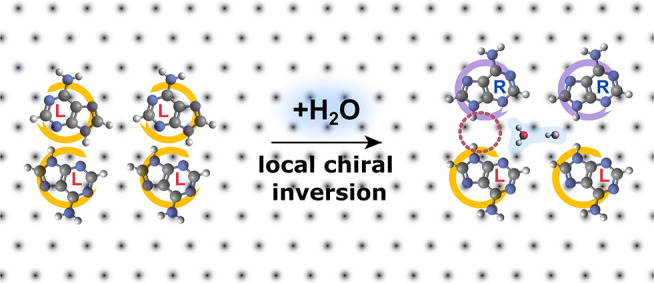
Schematic of the Adsorption Site Change, the Local Chiral Inversion, and the Counter-intuitive H-Bond “Mismatch” Induced by the Water Dimers in a Self-Assembled Adenine Layer Purple and orange colors represent left and right handedness, respectively. The underlaying substrate (Ag(111)) atomic lattice is indicated by the black dots.

A H-bonded supramolecular network of adenine molecules on Ag(111) (Figure 1a) is formed at RT under ultra-high vacuum (details in the Supporting Information (SI)). The self-assembled structure consists of horizontally aligned rows which are connected via H-bonds. The on-surface chirality^36^ of adenine can be distinguished in the enlarged high-resolution STM image (Figure 1b) with a CO-tip by their asymmetrically shaped structures. The corresponding constant-height AFM image (Figure 1c) further resolves the detailed structure of adenine molecules and their bonding network. The simulated AFM image (Figure 1d), with a superimposed DFT relaxed model of the canonical form of adenine, indicates that the adenine pair across the gap between rows is homochiral, though the whole network is racemic. The low-dimensional superstructure is stable at RT and resembles the bulk crystal structure of adenine,^37,38^ suggesting the intermolecular H-bonds are much stronger than the interaction with the substrate.

**Figure 1:**
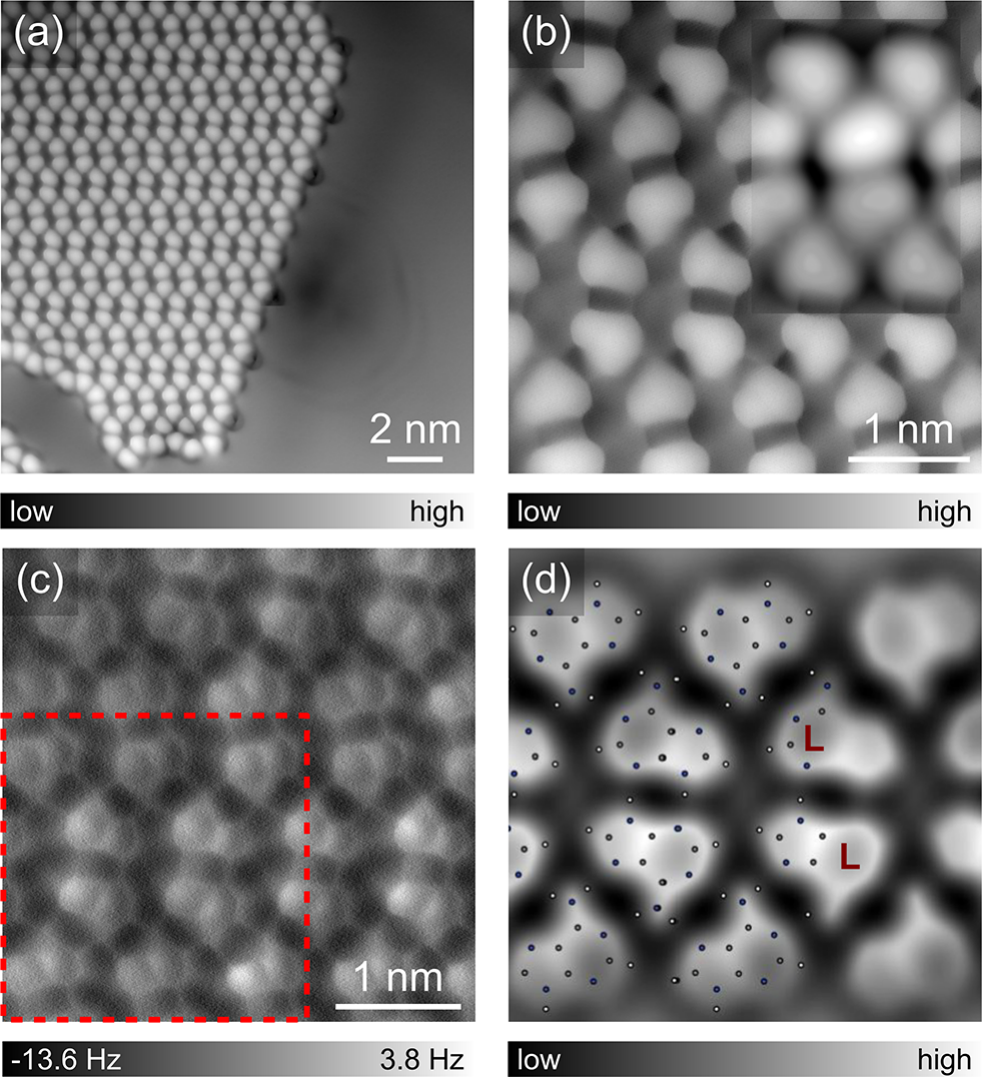
Self-assembly of adenine on Ag(111) probed by a CO-tip STM and AFM. (a) STM topography overview of an adenine island. Set point: 100 mV, 100 pA. (b, c) Zoomed-in STM topography of the island with a simulated STM image as an inset (b) and the corresponding constant-height AFM image (c). (d) Simulated AFM image with the overlaid molecular structure corresponding to the area marked in (c).

After exposure of the supramolecular network to water vapor at a pressure of ∼1 × 10^–5^ mbar for 15 min at a temperature of ∼200 K, some protrusions show up between rows of the superstructure in the high-resolution STM image (Figure 2a), originated from the insertion of water molecules.^39^ Here, the right “hole” of the superstructure contains a water molecule, and the simulated images correspond to this region. However, the constant-height AFM image looks similar to that of the structure without water (Figure 2a). The configuration of the adenine superstructure and the interstitial water revealed by the high-resolution STM image is matched in the simulated result (Figure 2b). In the inset of Figure 2b, the simulated AFM image based on the most reasonable configuration of the water molecule (see Figure S2) matches well with the experimental result. Also, a similar orientation of a single water molecule in an adenine superstructure has been previously reported.^26^

**Figure 2:**
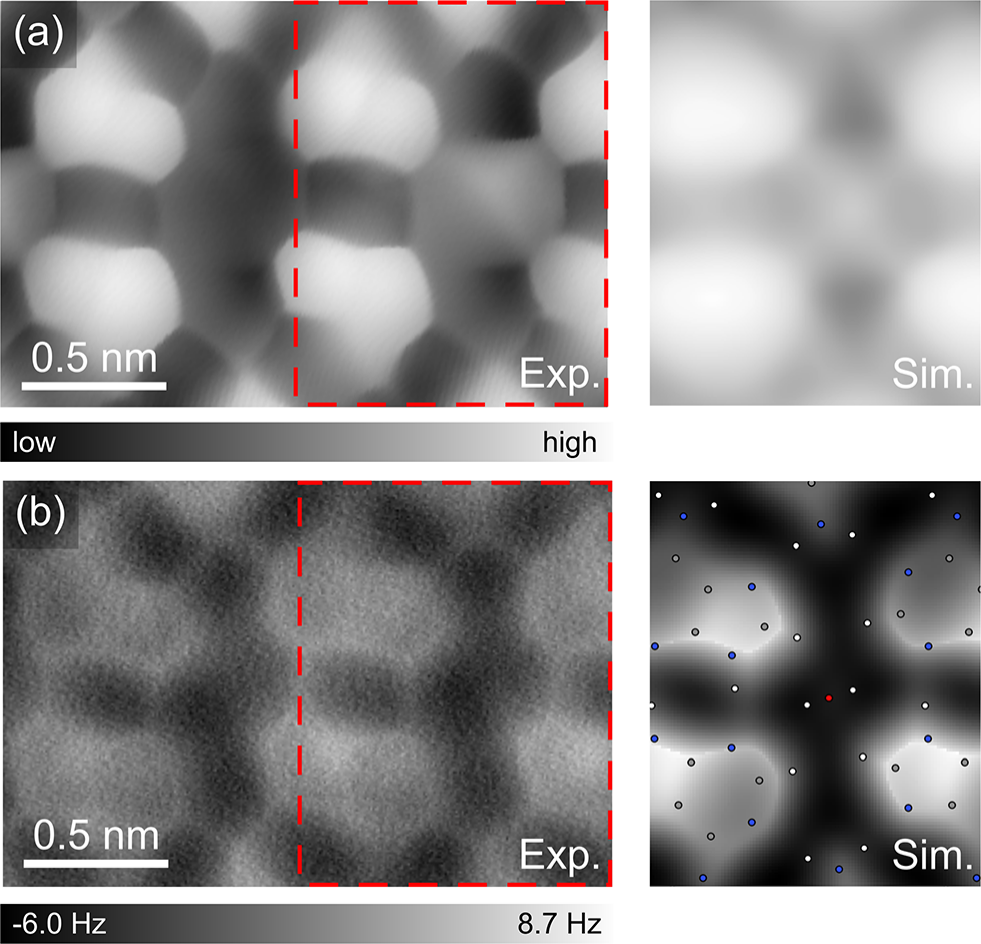
Adenine on Ag(111) after dosing with water molecules at low temperature. (a) STM topography showing water molecule(s) embedded among adenine molecules with a simulated STM image in the adjacent panel. Set point: 50 mV, 150 pA. Simulation done using 0.30 V bias. (b) The corresponding constant-height AFM image with a simulated AFM image in the adjacent panel. A DFT-optimized adsorbate structure is overlaid. Experimental images were recorded with a CO functionalized tip apex.

The very faint contrast of the single water molecule in the AFM image is presumably due to its position slightly lower than the adjacent adenine molecules. Overlaying the optimized structural model based on the DFT calculations (Figure 2b) reveals that the water molecule is stabilized by the surrounding adenine molecules through H-bonds and the surface chirality of adenines remains unchanged. The distance between rows increases very slightly (see Figures S7, S8, and S10), suggesting that introducing water has a minor effect on the layer when the substrate is at ∼200 K.

After water molecules are deposited on a sample that was held at RT, a water-involved rectangular superstructure, in contrast to the previous rhombic-shaped self-assembly, is obtained (see Figure S5). Also, some bright protrusion features and heterochiral adenine pairs across rows show up (Figure 3a). Noticeably, in the zoomed-in high-resolution STM image (Figure 3b), the bright protrusion feature surrounded by two adenine pairs closely resembles the STM image of water dimers reported earlier.^40^ The spatial arrangement of the heterochiral adenine pairs across rows indicates the conversion from a matched into a mismatched H-bonding pattern. The mismatched pattern is not found in the superstructure without water or with the water deposited when the substrate is at ∼200 K. In the constant-height AFM image, a dot-shaped protrusion shows up in the corresponding area of the bright protrusion in the STM image, and the distance between adenine rows is increased to about 0.82 nm (approximately 26% and 22% larger than those without water and with the water deposited when the substrate is at ∼200 K, respectively; see Figures S9 and S10). The simulated AFM image based on the proposed water dimer model with one flat and one upright water molecule agrees well with the experimental result, as shown in Figure 3c. As depicted in the optimized structure model (right part in Figure 3d), two N–H moieties in the adenine pairs point to each other and form an unusual mismatched H-bonding pattern between the molecules instead of the ordinary N–H···N bond.

**Figure 3:**
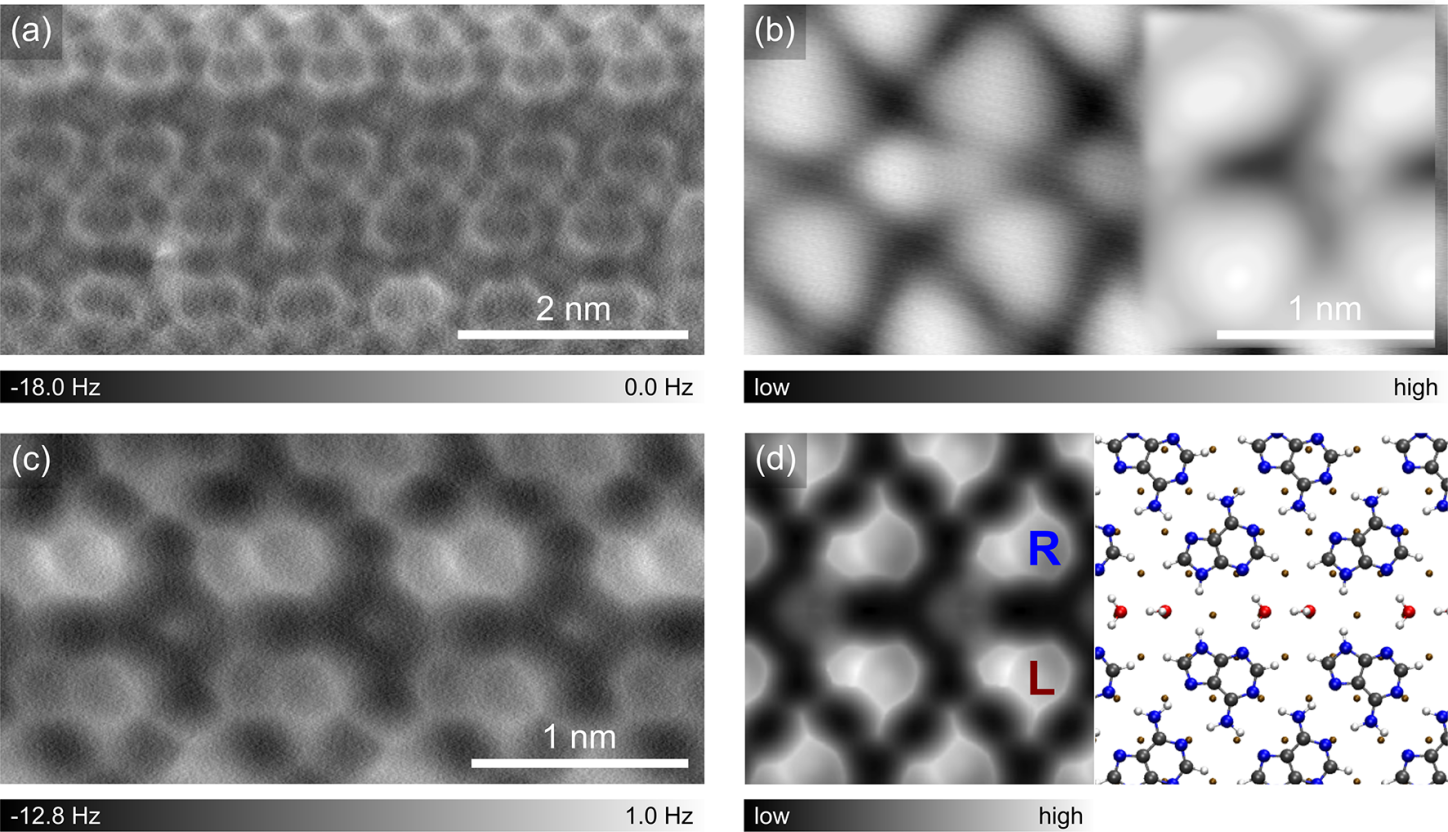
Annealed hydrated adenine structure on Ag(111) imaged with a CO-tip. (a) Constant-current AFM image of an adenine island with water. (b) Zoomed-in STM topography of the island with a simulated STM image as an inset. Set point: 250 mV, 100 pA. (c) Constant-height AFM image of the zoomed-in area of the island. (d) Simulated AFM image of the hydrated adenine structure and the corresponding molecular structure based on the DFT calculations.

Figure 4 compares the calculated adenine self-assembly structures with the usual H-bonding pattern as well as the mismatched H-bonding configuration with and without the presence of water dimers. The structure with linear non-planar (LNP) water dimers has the lowest energy. Other water dimer structures, e.g., a linear planar form where all four hydrogen atoms and two oxygen atoms lie in the same plane, have a higher total energy. Similarly to the LNP water dimer in our self-assembled structure, the linear non-planar water dimer is the minimum energy structure also in the gas phase.^41^ For the water dimer in the adenine layer, the O–H–O bond angle is 171.5°, and the H-bond is 1.81 Å in length (compared to 175.5° and 1.93 Å for an LNP dimer in the gas phase). The estimated energy gain from bonding to the water dimer, *E*_b_, before (NH···N) and after hydration (NH···N and OH···O) is −2.6 eV and −3.5 eV per unit cell, respectively, indicating that loss from the mismatched H-bonding is more than offset by the interaction with the water dimer (see SI, “Hydrogen-bonding energy gain”, page S-5). The energy difference between the mismatched hydrogen bond network with and without water dimers is approximately 1.2 eV per unit cell, clearly showing that a water dimer is necessary for stabilizing this unique structure. Finally, our experimental results and DFT calculations point to the presence of water dimers and not monomers in the self-assembled adenine layer. This observation is further supported by the fact that water dimers are predicted to be better hydrogen donors and acceptors compared to water monomers,^42^ which also implies stronger interactions between the dynamically self-assembled layer and the spontaneously formed water dimers.

**Figure 4:**
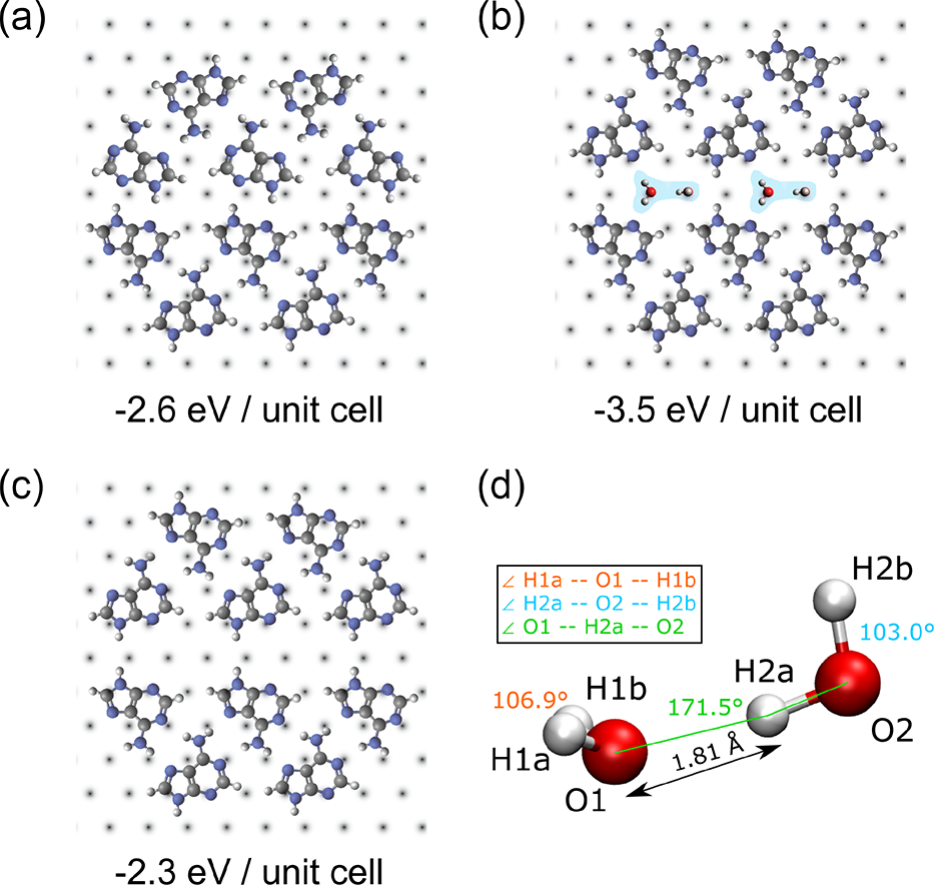
(a–c) Structural models of the self-assembled adenine structures without (a, c) and with water dimers (b). Panel (a) shows the adenine layer with the H-bond matched structure, while panels (b) and (c) depict the H-bond mismatched structure. (d) Detailed structure of the linear non-planar water dimer found in the adenine layer.

To rule out the possible tautomeric behavior of adenine in the self-assembled layer, we performed further DFT calculations and AFM simulations on possible structures, as previous research indicates that other tautomers besides the canonical form might appear at RT in a micro-hydrated environment.^43^ However, we found no concrete evidence suggesting that secondary tautomers are found in the hydrated supramolecular network, and the canonical form adenine molecule should be the primary component.

In conclusion, by introducing water to the self-assembled adenine layers at RT, individual water dimers were formed and stabilized inside the restructured adenine 2D network. With STM, AFM, and DFT simulations, we successfully revealed the detailed bonding structure of the confined water dimers as well as the re-arrangement the local adenine network undergoes upon hydration. The water dimers under confinement appear to be in a linear non-planar configuration, causing a local chirality inversion such that a distinctive mismatched H-bond pattern emerged between neighboring adenine molecules. The comprehensive characterization of the ensemble of water dimers and adenine not only provides crucial insights into the dynamic nature of the hydration process of DNA bases but also offers a novel method to study unstable small-molecule clusters which would otherwise be impossible to observe.

## Supporting Information Available

Experimental and computational methods; additional experimental results and details of the determination of the lattice parameters of the self-assembled layers, including Figures S1–S10 (PDF)
