# Supplementary material for: Water Dimer-Driven DNA Base Superstructure with Mismatched Hydrogen Bonding
Source: J Am Chem Soc. 2022 Oct 27;144(44):20227–31. doi: 10.1021/jacs.2c09575 (PMC9650709; doi:10.1021/jacs.2c09575)
Supplement: Supplementary file 1 — ja2c09575_si_001.pdf [file ja2c09575_si_001.pdf]

Supporting Information: Water dimer driven DNA base superstructure with mismatched hydrogen-bonding

Shuning Cai,[†,¶] Lauri Kurki,[†,¶] Chen Xu,[†,¶] Adam S. Foster,[*,†,‡] and Peter Liljeroth[*,†]

†*Department of Applied Physics, Aalto University, 00076 Aalto, Espoo, Finland*

‡*WPI Nano Life Science Institute (WPI-NanoLSI), Kanazawa University, Kakuma-machi, Kanazawa 920-1192, Japan*

¶*These authors contributed equally.*

E-mail: adam.foster@aalto.fi; peter.liljeroth@aalto.fi

Methods

Experimental

The experiments were carried out on a combined non-contact AFM/STM system (CreaTec) with a commercial qPlus sensor with a Pt/Ir tip, operating at $T \approx 5$ K in ultrahigh vacuum at a pressure of $\sim 1 \times 10^{-10}$ mbar. The qPlus sensor had a resonance frequency of $f_0 \approx 30046$ Hz, a quality factor $Q \approx 67714$, and was always operating with an oscillation amplitude of $A = 50$ pm.

The Ag(111) substrate (MaTeck) was prepared by repeated Ne^+ sputtering with a beam energy of 1000 eV and ion current of 30 µA for 15 min followed by annealing at $\sim 480-500$°C

for 5 min. A flat Ag(111) surface with large terrace and minimum amount of impurities was obtained within 3 cycles. The adenine molecules (Sigma-Aldrich; purity $\geq$ 99%) were deposited onto the substrate at $\sim$ 5 K through thermal sublimation at 120°C at a chamber pressure of 1×10^{-6} mbar for 15 min. A self-assembly layer of adenine can be formed by slightly warming up of the substrate for 5 min. Then the CO molecules (Praxair; purity 99.997%) were deposited onto the substrate at $\sim$ 5 K through a variable leak valve at a chamber pressure of 1×10^{-8} mbar for 80 seconds.

Before deposition, the adenine was thoroughly degassed at 120°C for 1 h. While the water was firstly boiled at 100°C to rid of any residual gas inside, and was then degassed thoroughly via several freeze-pump-thaw cycles.[S1]

During the hydration process, the sample was held at $T \approx 200$ K or room temperature, and then was transferred to the preparation chamber. The water molecule (Sigma-Aldrich SKU38796; deionized) was introduced into the preparation chamber through a variable leak valve aiming directly at the sample. The pressure was maintained at $\sim 1 \times 10^{-5}$ mbar for 15 min. The sample was later transferred back to the main chamber and cooled down to 5 K for imaging.

Computational

DFT calculations. All calculations were performed using the all-electron density functional theory code FHI-AIMS[S2,S3] with the PBE exchange correlation functional[S4] and the Tkatchenko-Scheffler method[S5] was used to accurately handle the van der Waals interactions. The basis sets as defined in FHI-AIMS were used at the "light" level for all atoms. A trust radius enhanced version of the BFGS algorithm was used to relax the structures to a force less than 10^{-2} eV/Å. To acquire an approximately homogeneous k-grid in the plane parallel to the substrate, a $7 \times 2 \times 1$-grid was used.

As Fig. S1 shows, the adenine monolayer was modeled as a pair of adenine chains that are periodic only in the horizontal direction. Still, one gap across the chains is retained and

the simulated AFM images in this region show good correspondence to the experimentally acquired images. The choice to exclude the periodicity in the vertical direction was made as there was a significant lattice mismatch between the Ag(111) substrate and the adsorbed adenine monolayer. In the horizontal direction, the lattice mismatch was less than 2 % in all structures. As the inclusion of water mostly affected the vertical gap between the chains and not the horizontal, we could use the same unit cell dimensions in all calculations. Three substrate layers were used in all calculations. The DFT relaxed hydrated layers modelling the experiments at $T = 200\,\text{K}$ and at room temperature are shown in Fig. S2 and Fig. S3 respectively.

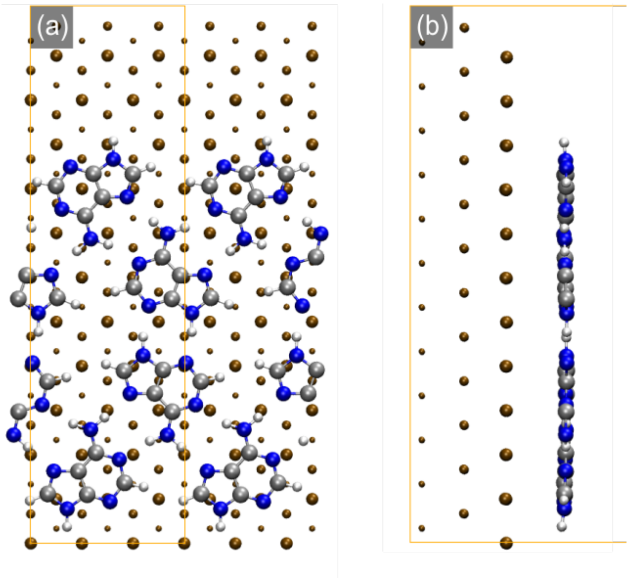

Figure S1: (a) The DFT relaxed structure of the pure A layer in top view with one unit cell ($8.63\,\text{Å} \times 29.9\,\text{Å} \times 30.0\,\text{Å}$) highlighted. The three layers of the substrate are illustrated with spheres of different radii. (b) The relaxed structure in side view.

AFM simulations. The probe particle model[S6,S7] was used to produce the simulated AFM images. To emulate the behaviour of the CO tip, we used a lateral spring constant of 0.24 N/m and a radial spring constant of 20.00 N/m for the probe particle. Further, the tip charge was modeled as a quadrupole with a quadrupole moment of $-0.05\,e \times \text{Å}^2$. These are standard parameters for CO tip imaging.[S6,S7] The electrostatic field of the sample was obtained using FHI-AIMS.

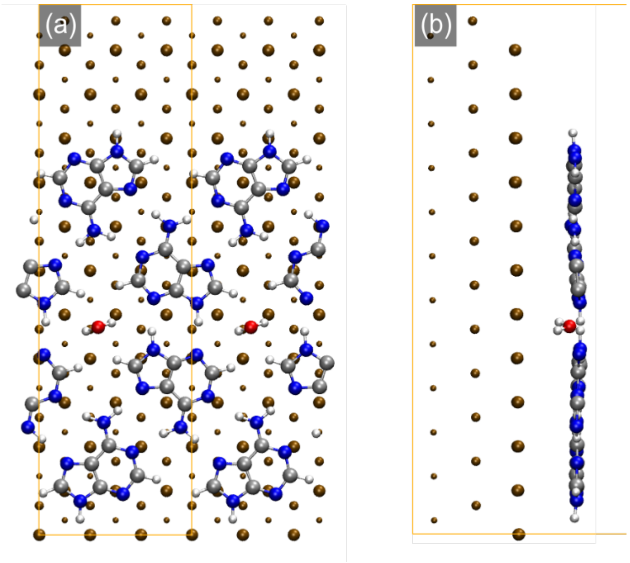

Figure S2: (a) The DFT relaxed structure of the hydrated A layer modelling experiments at $T \approx 200\,\mathrm{K}$ in top view with one unit cell highlighted. (b) The relaxed structure in side view.

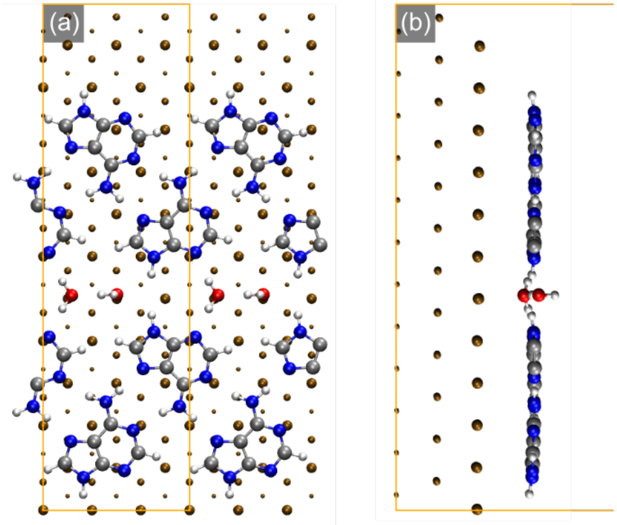

Figure S3: (a) The DFT relaxed structure of the hydrated A layer modelling experiments at room temperature in top view with one unit cell highlighted. (b) The relaxed structure in side view.

STM simulations. All simulated STM images were obtained using the PP-STM code.[S8] The scans were performed using the relaxed tip positions acquired during the AFM simulation. The broadening factor was set at 0.5 eV and standard CO tip orbital parameters were used with 13% s orbital and 87% p_{xy} orbital contributions.[S9] The constant-current images

were produced by calculating an isosurface from a stack of constant-height images at an isovalue of approximately 10% of the maximum current. For all structures, the constant-height stack was calculated between 5 Å and 7 Å above the sample. A 0.1 Å spacing was used for the constant-height scans.

Hydrogen-bonding energy gain. As the number of atoms in the structures change, we cannot directly compare the total energies of the structures. Instead, we calculate and compare the so called hydrogen-bonding energy gain per unit cell which describes the energy in the adenine – adenine and adenine – H_2O hydrogen-bonds in the adsorbed layer. We define the energy gain as $E_{\text{gain}} = E_{\text{total}} - E_{\text{slab}} - 4E_{\text{adenine}} - 4E_{\text{adsorption}}$, where $E_{\text{adsorption}}$ is the adsorption energy of a single adenine molecule, E_{adenine} is the energy of a single isolated adenine molecule, and E_{slab} is the energy of the substrate calculated using a slab including the adsorbed water molecule(s). Each of the unit cells contains 4 adenine molecules. We include the water molecules in the slab to exclude the H_2O – H_2O hydrogen bonding from the energy comparisons.

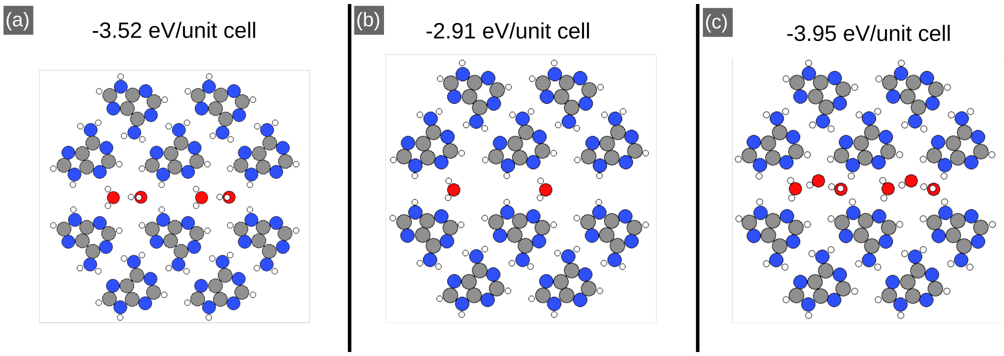

Figure S4: Three DFT relaxed adenine superstructures with the corresponding hydrogen-bonding energy gains: (a) dimer, (b) monomer and (c) trimer.

Fig. S4 shows the hydrogen-bonding energy gains for the dimer model and DFT relaxed trimer and monomer configurations for comparison. These calculations support the dimer argument in the adenine self-assembly – the superstructures with a water monomer have a higher energy compared to the dimer configuration. More specifically, the H-bonding energy gain per unit cell for the monomer case is -2.91 eV and it increases to -3.52 eV with the

dimer and to -3.95 eV with the trimer. That is, the increase we see from monomer to dimer is -0.61 eV and from dimer to trimer it is -0.43 eV. This means that an available water molecule rather forms a dimer with an existing monomer than a trimer with an existing dimer. Further, the prediction that the dimer configuration is energetically favourable is supported by much better agreement between simulated SPM images and experiments in comparison to the monomer and trimer.

Overview STM images

Fig. S5 shows large scale overview images of the adenine self-assembly structure for non-hydrated and RT-annealed hydrated samples.

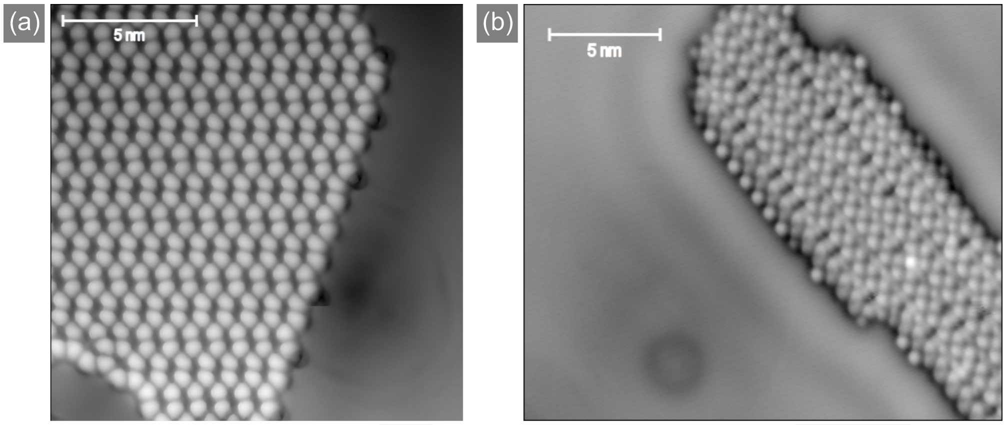

Figure S5: STM images with CO-tip showing large overview of (a) a non-hydrated assembly and (b) a hydrated adenine assembly after annealing at RT.

The individual water molecules at 200 K

Fig. S6 shows the zoomed-in imaging on the monomer water in-between the adenine assembly after annealing at 200K. The STM shows a protrusion for the monomer water molecule, while the constant current nc-AFM cannot resolve the monomer water molecule, because the oxygen of the water molecule is located approximately 0.6 Å below the adenine layer.

AFM images under constant-current mode are perhaps not the optimal mode for detecting the water molecules here as the feedback retracts the tip over the expected position of the water molecule (as it appears as a protrusion in the STM).

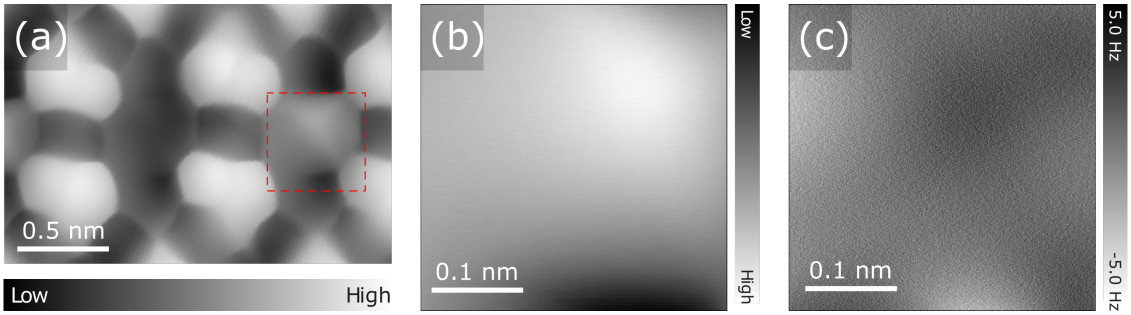

Figure S6: STM topography (b) and constant-current AFM (c) (Setpoint: 50 mV, 200 pA) of the protrusion area in the STM topography of the adenine self-assembly at 200 K (a) marked by the red dashed box.

Determination of the lattice parameters of the adenine structures

We have used line profile analysis of the STM images to determine the spacing between the adenine rows in the three different samples (non-hydrated, hydrated and annealed to 200 K, and hydrated and annealed to RT), see Figs. S7-S9. The analysis can be complemented by using 2D autocorrelation as shown in Fig. S10, which allows determining the lattice parameters of the structures. This can be converted to the distance between the pairs of adenines as indicated in the figure.

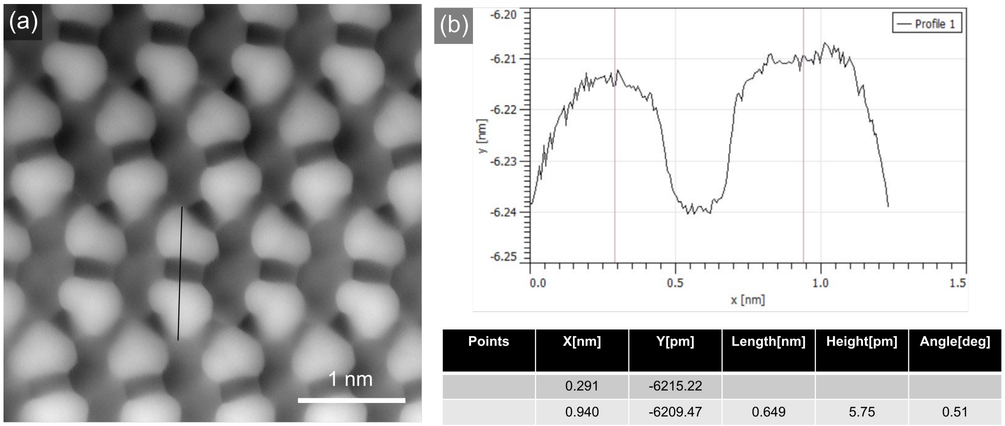

Points	X[nm]	Y[pm]	Length[nm]	Height[pm]	Angle[deg]
	0.291	-6215.22			
	0.940	-6209.47	0.649	5.75	0.51

Figure S7: Example of the distance determination between the adenine rows before hydration. Average over several line profiles yields 0.65 nm.

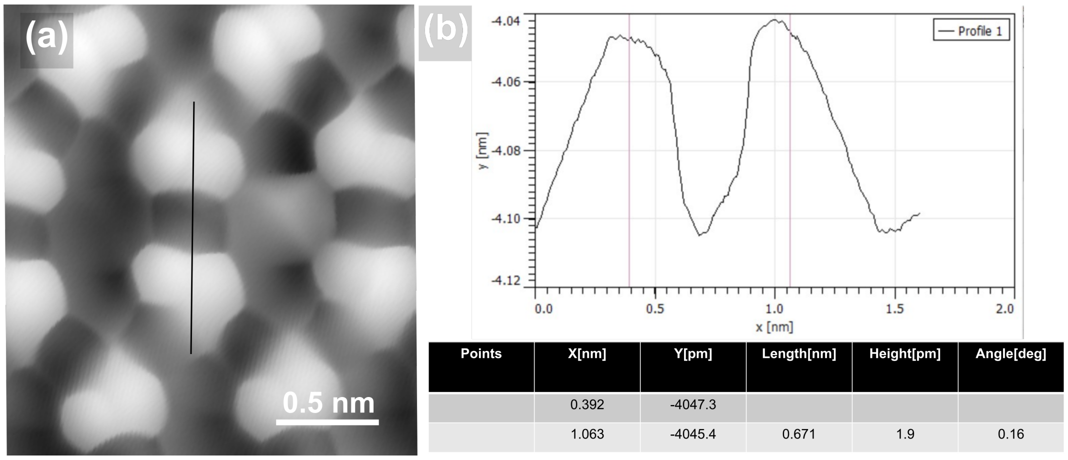

Points	X[nm]	Y[pm]	Length[nm]	Height[pm]	Angle[deg]
	0.392	-4047.3			
	1.063	-4045.4	0.671	1.9	0.16

Figure S8: Example of the distance determination between the adenine rows after hydration at 200 K. Average over several line profiles yields 0.67 nm.

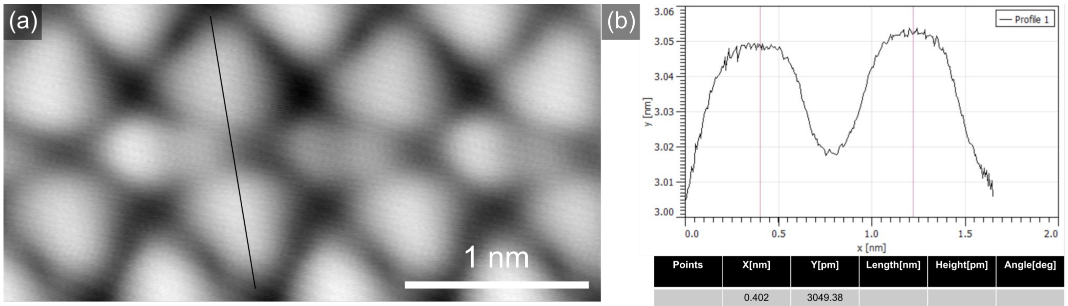

Points	X[nm]	Y[pm]	Length[nm]	Height[pm]	Angle[deg]
	0.402	3049.38			
	1.222	3051.87	0.820	2.49	0.17

Figure S9: Example of the distance determination between the adenine rows after hydration at RT. Average over several line profiles yields 0.82 nm.

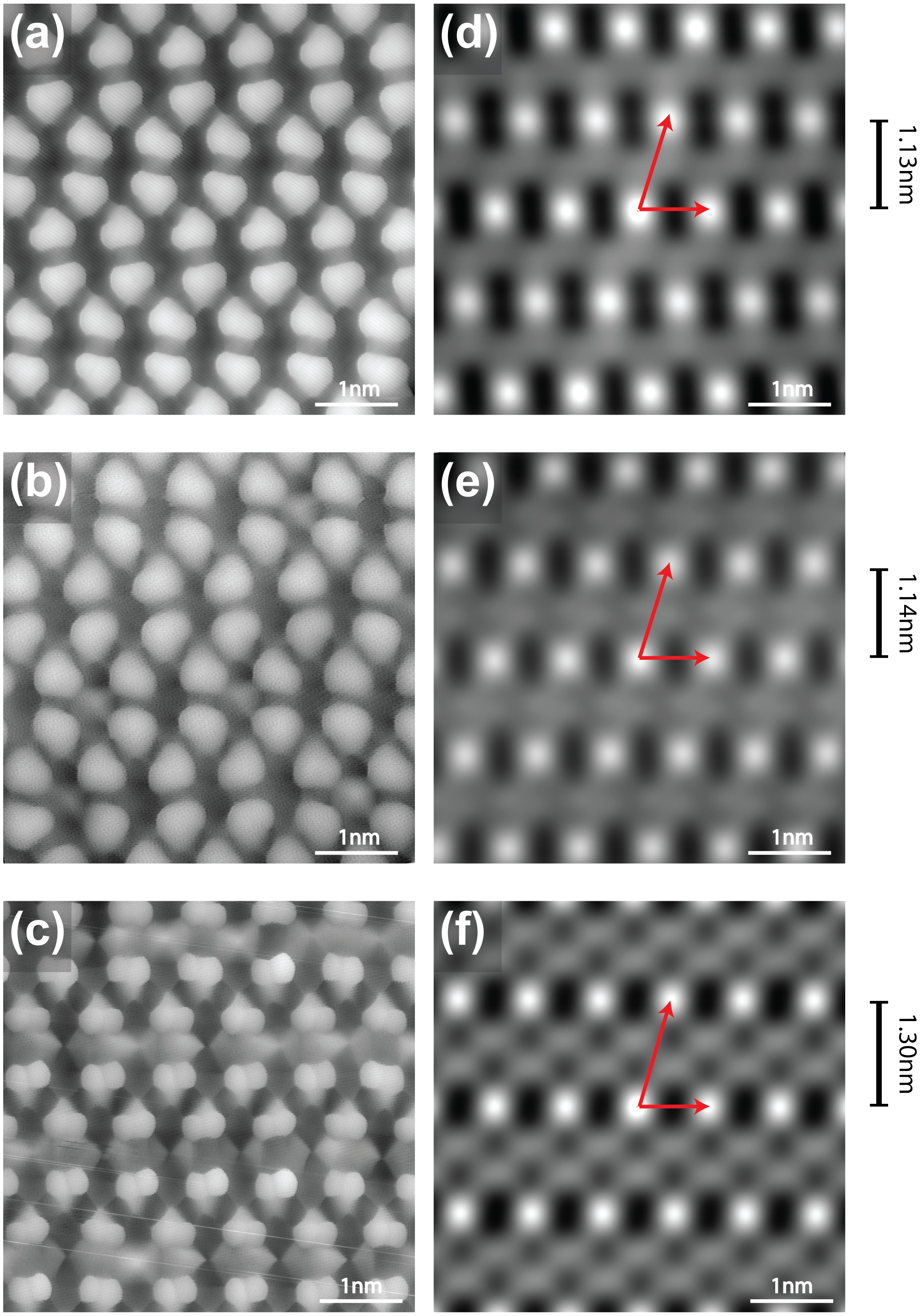

Figure S10: Measuring the lattice parameter of the self-assembled adenine structures. (a), (b) and (c) STM topography with CO-tip showing adenine layer without water, after hydration at 200K and after hydration at RT, respectively. (d) 2D auto-correlation[S10] of (a), the distance between un-hydrated adenine rows is 1.13 nm, which is calculated from the lattice of the 2D auto-correlation, marked by the red arrows. Similarly for (e) and (f), the distance for adenine rows after hydration at 200K is 1.14 nm and for after hydration at RT, the distance becomes 1.30 nm.

References

(S1) Oinonen, N.; Xu, C.; Alldritt, B.; Canova, F. F.; Urtev, F.; Cai, S.; Krejčí, O.; Kannala, J.; Liljeroth, P.; Foster, A. S. Electrostatic Discovery Atomic Force Microscopy. *ACS Nano* **2022**, *16*, 89–97.

(S2) Havu, V.; Blum, V.; Havu, P.; Scheffler, M. Efficient O(N) integration for all-electron electronic structure calculation using numeric basis functions. *J. Comput. Phys.* **2009**, *228*, 8367–8379.

(S3) Blum, V.; Gehrke, R.; Hanke, F.; Havu, P.; Havu, V.; Ren, X.; Reuter, K.; Scheffler, M. Ab initio molecular simulations with numeric atom-centered orbitals. *Comput. Phys. Commun.* **2009**, *180*, 2175–2196.

(S4) Perdew, J. P.; Burke, K.; Ernzerhof, M. Generalized Gradient Approximation Made Simple. *Phys. Rev. Lett.* **1996**, *77*, 3865–3868.

(S5) Tkatchenko, A.; Scheffler, M. Accurate molecular van der Waals interactions from ground-state electron density and free-atom reference data. *Phys. Rev. Lett.* **2009**, *102*, 073005.

(S6) Hapala, P.; Kichin, G.; Wagner, C.; Tautz, F. S.; Temirov, R.; Jelínek, P. Mechanism of high-resolution STM/AFM imaging with functionalized tips. *Phys. Rev. B* **2014**, *90*, 085421.

(S7) Hapala, P.; Temirov, R.; Tautz, F. S.; Jelínek, P. Origin of High-Resolution IETS-STM Images of Organic Molecules with Functionalized Tips. *Phys. Rev. Lett.* **2014**, *113*, 226101.

(S8) Krejčí, O.; Hapala, P.; Ondráček, M.; Jelínek, P. Principles and simulations of high-resolution STM imaging with a flexible tip apex. *Phys. Rev. B* **2017**, *95*, 045407.

(S9) De La Torre, B.; Švec, M.; Foti, G.; Krejčí, O.; Hapala, P.; Garcia-Lekue, A.; Frederiksen, T.; Zbořil, R.; Arnau, A.; Vázquez, H.; Jelínek, P. Submolecular Resolution by Variation of the Inelastic Electron Tunneling Spectroscopy Amplitude and its Relation to the AFM/STM Signal. *Phys. Rev. Lett.* **2017**, *119*, 166001.

(S10) Nečas, D.; Klapetek, P. Gwyddion: an open-source software for SPM data analysis. *Open Phys.* **2012**, *10*, 181–188.
